# Thymic Stromal Lymphopoietin Isoforms, Inflammatory Disorders, and Cancer

**DOI:** 10.3389/fimmu.2018.01595

**Published:** 2018-07-13

**Authors:** Gilda Varricchi, Antonio Pecoraro, Giancarlo Marone, Gjada Criscuolo, Giuseppe Spadaro, Arturo Genovese, Gianni Marone

**Affiliations:** ^1^Department of Translational Medical Sciences and Center for Basic and Clinical Immunology Research, University of Naples Federico II, Naples, Italy; ^2^WAO Center of Excellence, Naples, Italy; ^3^Department of Public Health, University of Naples Federico II, Naples, Italy; ^4^Monaldi Hospital Pharmacy, Naples, Italy; ^5^Institute of Experimental Endocrinology and Oncology “Gaetano Salvatore”, National Research Council (CNR), Naples, Italy

**Keywords:** asthma, atopic dermatitis, cancer, inflammation, thymic stromal lymphopoietin

## Abstract

Thymic stromal lymphopoietin (TSLP) is a pleiotropic cytokine originally isolated from a murine thymic stromal cell line. TSLP exerts its biological effects by binding to a high-affinity heteromeric complex composed of thymic stromal lymphopoietin receptor chain and IL-7Rα. TSLP is primarily expressed by activated lung and intestinal epithelial cells, keratinocytes, and fibroblasts. However, dendritic cells (DCs), mast cells, and presumably other immune cells can also produce TSLP. Different groups of investigators have demonstrated the existence of two variants for TSLP in human tissues: the main isoform expressed in steady state is the short form (sf TSLP), which plays a homeostatic role, whereas the long form (lfTSLP) is upregulated in inflammatory conditions. In addition, there is evidence that in pathological conditions, TSLP can be cleaved by several endogenous proteases. Several cellular targets for TSLP have been identified, including immune (DCs, ILC2, T and B cells, NKT and Treg cells, eosinophils, neutrophils, basophils, monocytes, mast cells, and macrophages) and non-immune cells (platelets and sensory neurons). TSLP has been originally implicated in a variety of allergic diseases (e.g., atopic dermatitis, bronchial asthma, eosinophilic esophagitis). Emerging evidence indicates that TSLP is also involved in chronic inflammatory (i.e., chronic obstructive pulmonary disease and celiac disease) and autoimmune (e.g., psoriasis, rheumatoid arthritis) disorders and several cancers. These emerging observations greatly widen the role of TSLP in different human diseases. Most of these studies have not used tools to analyze the expression of the two TSLP isoforms. The broad pathophysiologic profile of TSLP has motivated therapeutic targeting of this cytokine. Tezepelumab is a first-in-class human monoclonal antibody (1) that binds to TSLP inhibiting its interaction with TSLP receptor complex. Tezepelumab given as an add-on-therapy to patients with severe uncontrolled asthma has shown safety and efficacy. Several clinical trials are evaluating the safety and the efficacy of tezepelumab in different inflammatory disorders. Monoclonal antibodies used to neutralize TSLP should not interact or hamper the homeostatic effects of sf TSLP.

## Introduction

Thymic stromal lymphopoietin (TSLP) is a pleiotropic cytokine originally isolated from a murine thymic stromal cell line (2) and characterized as a lymphocyte growth factor (3). A human homolog was identified using *in silico* methods (4, 5). The human TSLP gene is located on chromosome 5q22.1 next to the atopic cytokine cluster on 5q31 (6), while the murine *Tslp* is mapped on chromosome 18 (3). Human and mouse TSLP exert their biological activities by binding to a high-affinity heteromeric complex composed of thymic stromal lymphopoietin receptor (TSLPR) chain and interleukin 7 receptor-α (IL-7Rα) (7, 8). The sequence homology between mouse and human TSLP is only about 40%, and their biological functions are similar (5), but not identical (9). TSLP is expressed predominantly by gut (10–15) and lung epithelial cells (16–21), skin keratinocytes (15, 22–25), and by dendritic cells (DCs) (26). However, TSLP can be produced also by airway smooth muscle cells (27), human DCs (26) and mast cells (16, 25, 28, 29), human monocytes (26), macrophages and granulocytes (30), synovial (31) and cancer-associated fibroblasts (CAF) (32), murine basophils (33), and cancer cells (34).

For many years, TSLP has been widely studied in the regulation of inflammatory processes occurring at the barrier surfaces (e.g., skin, lung, and gut). In fact, TSLP activates TSLPR^+^ DCs and plasmacytoid DCs to induce functional Th2 and regulatory T (Treg) cells (35, 36) and human T follicular helper (Tfh) cells (37). Interestingly, TSLP has now emerged as a cytokine with a plethora of pleiotropic properties mediated by the activation of a broad range of immune and non-immune cells. Depending on the immune cells targeted by TSLP, it is reported not only to promote Th2 response but also to be associated with autoimmune disorders (38–40) and cancer (32, 34, 41). Such a broad pathophysiological profile has motivated therapeutic targeting of TSLP- and TSLPR-mediated signaling (42–45).

### TSPL—TSLP Receptor Interaction

**Figure 1:**
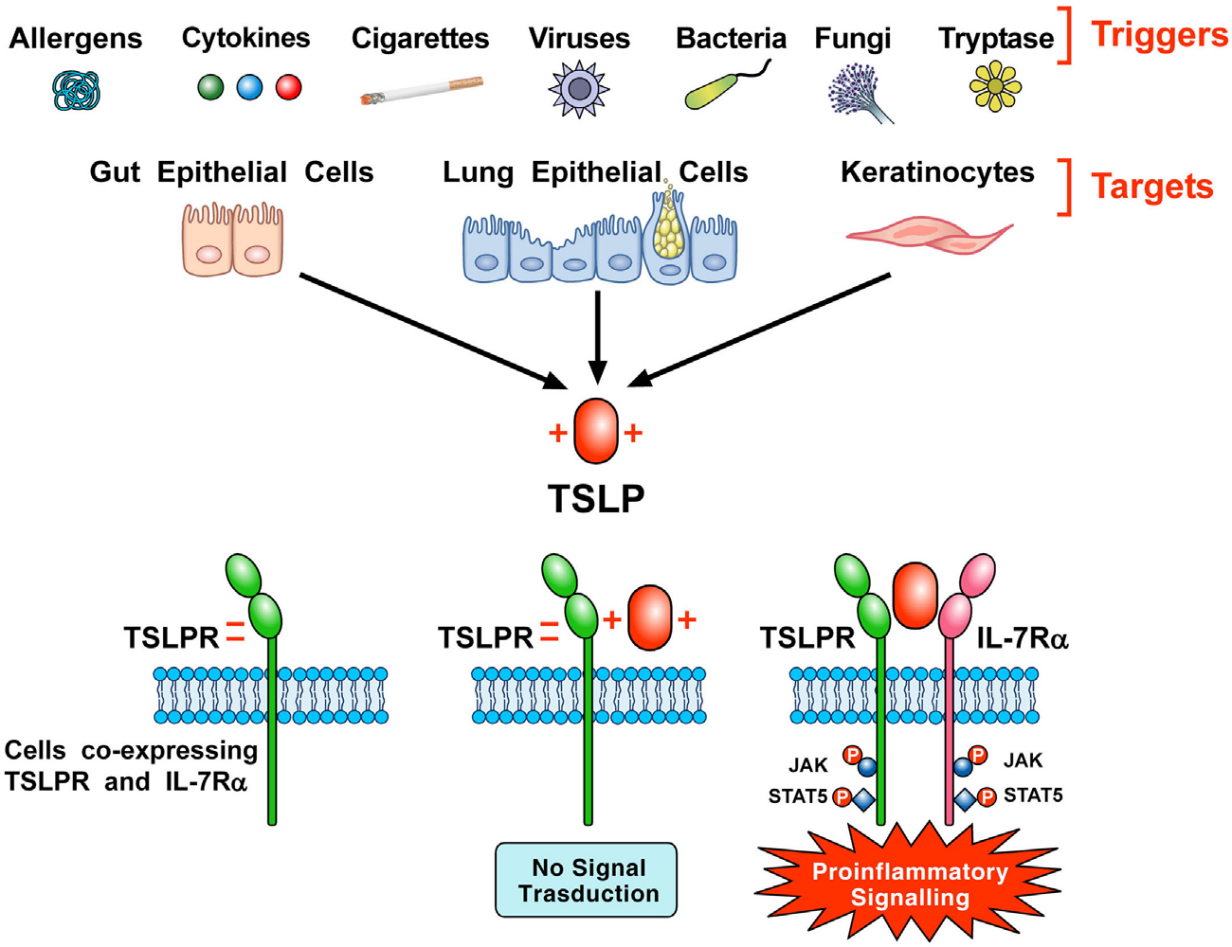
Schematic representation of the production of thymic stromal lymphopoietin (TSLP) and its signaling complex *via* a cooperative stepwise mechanism on the surface of cellular targets. A plethora of triggers including allergens, cigarette smoke extracts, cytokines, viral, bacterial and fungal products, and tryptase can activate lung and gut epithelial cells and keratinocytes to release TSLP. The latter, which is positively charged, binds to thymic stromal lymphopoietin receptor (TSLPR), which is negatively charged, with high affinity and fast kinetics. Then, IL-7Rα associates with performed TSLPR:TSLP binary complex to form the ternary TSLPR-TSLP-IL-7Rα complex (44). This receptor complex on cells co-expressing TSLPR and IL-7Rα phosphorylates JAK and STAT5 to initiate proinflammatory signaling.

Thymic stromal lymphopoietin initiates signaling by establishing a ternary complex with its specific receptor, TSLPR, and then with IL-7Rα (7, 8). The latter receptor also serves, together with the common γ-chain (γc) receptor, in signaling complex driven by IL-7 to modulate T cell development (46). First human TSLP, positively charged, binds to TSLPR, which is negatively charged, with high affinity and fast kinetics (44). Then, IL-7Rα binds to the preformed TSLP:TSLPR binary complex with high affinity. The formation of the ternary complex, TSLPR:TSLP: IL-7Rα, initiates signaling in cells co-expressing TSLPR and IL-7Rα (Figure 1). The variable heavy chain of human monoclonal antibody (mAb) anti-TSLP, tezepelumab, binds to TSLP, while the variable light chain fragment does not interact with TSLP (44). Tezepelumab inhibits *in vitro* human blood DC maturation and chemokine (CCL17) production induced by TSLP (44).

### Activators of TSLP Production

Several cytokines (e.g., TNF-α, IL-1β) (13, 14, 16, 20), respiratory viruses (17–19, 21, 47), bacterial (e.g., *Staphylococcus aureus*) (23) and fungal products (48), mechanical injury (49), allergens (50), cigarette smoke extracts (51, 52), and tryptase (53) can induce the expression of TSLP from different target cells.

## Cellular Targets and Biological Properties of TSLP

**Figure 2:**
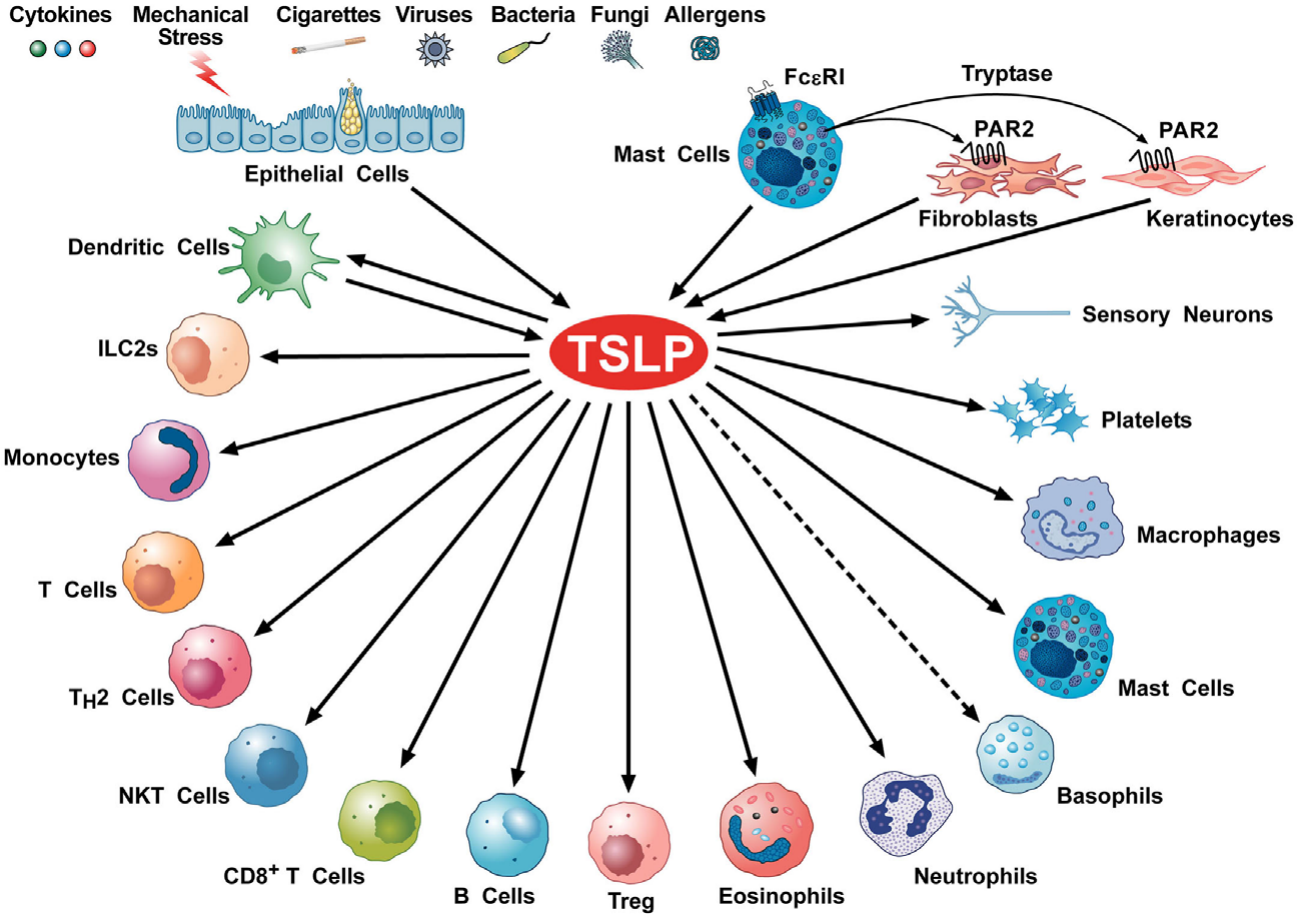
Schematic representation of cellular targets of thymic stromal lymphopoietin (TSLP). Several triggers can activate lung and gut epithelial cells and keratinocytes to release TSLP. This cytokine can also be produced by activated mast cells (28, 55–57) and dendritic cells (DCs) (26, 58). Tryptase, released by mast cell activates the protease-activated receptor 2 receptor on fibroblasts (53, 59) and keratinocytes (53) to release TSLP. TSLP activates DCs (37, 44, 60), ILC2 (61–63), CD4^+^ T and Th2 cells (64, 65), NKT cells (66), CD8^+^ T cells (67) and B cells (68, 69), Treg (35, 70), eosinophils (71, 72), neutrophils (73), murine (74), but not human basophils (9), monocytes (75), mast cells (16, 76–78), macrophages (79), platelets (80, 81), and sensory neurons (53).

Several cellular targets of TSLP have been identified, including immune and non-immune cells (Figure 2). DCs have a critical role in promoting Th2 cytokine responses (54). TSLP-stimulated DCs activate CD4^+^ T cells. Culture of TSLP-activated DCs together with naive syngeneic CD4^+^ T cells leads to T cell proliferation, which suggests a role for TSLP in CD4^+^ T cell homeostasis. However, when TSLP-stimulated DCs prime CD4^+^ T cells in an antigen-specific manner, the resulting T cells show characteristic features of T helper type 2 (Th2)-differentiated cells (production of IL-4, IL-5, and IL-13) (25). These data suggest that TSLP-activated DCs prime for inflammatory Th2 cell differentiation (32).

In addition to its effects on the differentiation of CD4^+^ Th2 cells potentially *via* DCs and/or basophils (32), TSLP is able to directly promote the Th2 cell differentiation of naive T cells. The combination of TCR stimulation and TSLP treatment can induce IL-4 transcription and Th2 differentiation (64).

Type-2 immune responses that occur in airways of asthmatics are accompanied by goblet cell hyperplasia and mucus production and are driven by IL-33, TSLP, and IL-25 produced by activated lung epithelial cells (16, 82, 83). These three cytokines target members of group 2 innate lymphoid cells (ILC2s) (61, 84, 85). TSLP is a critical mediator acting on ILC2s in allergen-induced airway inflammation (61, 62) and drives the development of Th2 cells (65, 86) and CD4^+^ T cells (87). TSLP also provides critical signals for human B cell proliferation (68), Treg expansion (36, 70), and Tfh differentiation (37).

Human peripheral blood monocytes can be divided into three major subsets, classical, intermediate, and non-classical monocytes based on their expression of CD14 and CD16 [CD14^high^ CD16^−^, CD14^+^ CD16^+^, and CD14^dim^ CD16^+^, respectively (88)]. It was originally demonstrated that *TSLPR* and *IL-7R*α mRNAs are coexpressed in human monocytes and that TSLP induces the production of CCL17 (5). Borriello and co-workers recently demonstrated that freshly isolated monocytes do not express TSLPR and IL-7Rα by flow cytometry, nor they phosphorylate STAT5 in response to TSLP (75). However, stimulation of monocytes with lipopolysaccharide (LPS) induced the expression of TSLPR complex on a small percentage of human monocytes. TSLP enhanced CCL17 production by monocytes induced by LPS and IL-4. The *in vivo* relevance of this original observation is supported by the demonstration that monocytes from patients with Gram-negative sepsis have a higher expression of *TSLPR* and *IL-7R*α mRNAs compared to healthy controls (89). The elegant study by Borriello and collaborators unravels a previously unknown phenotypic and functional heterogeneity of human monocytes based on TSLPR expression.

Several groups have reported that TSLP activates human eosinophils through the engagement of TSLPR and IL-7Rα expressed on their surface (71, 72, 90, 91). Recently, it has been reported that TSLP acts on mouse and human neutrophils to enhance *S. aureus* killing in a complement-dependent manner (73).

The relevance of TSLP–TSLPR axis and basophils has been emphasized in several experimental models. Siracusa and collaborators reported that TSLP promotes peripheral basophilia and that TSLPR-expressing basophils can restore Th2-dependent immunity in mice (74). This study was followed by the observation that a mouse model of eosinophilic esophagitis (EoE)-like disease was dependent on TSLP acting on basophils (92). In the same study, the authors reported overexpression of *TSLP* and increased basophil responses in esophageal biopsies of EoE patients. The group of Gauvreau found that approximately 10% human basophils express TSLPR and that TSLP increases histamine release from basophils (93). By contrast, a collaborative study demonstrated that basophils from allergic patients do not respond to TSLP and do not express IL-7Rα (9). It is well established that human basophils differ from mouse basophils (94), and these differences might explain, at least in part, the different effects of TSLP on human and mouse basophils.

Thymic stromal lymphopoietin–TSLPR interactions appear essential for immunity to *Trichuris* (95, 96). The importance of the TSLP pathway and basophils in protective immunity to *Trichuris*, coupled with the demonstration that delivery of recombinant TSLP can augment basophil numbers in the periphery (97), suggests that coordinated TSLP-mediated regulation of DCs and basophils may have an important role in developing Th2 cytokine responses.

The expression of both TSLPR and IL-7Rα has been reported in CD34^+^ progenitor mast cells and human lung mast cells at the transcript and protein level (16). TSLP, alone or in combination with IL-1β or TNF-α, does not induce mast cell degranulation or lipid mediator release (16, 55). However, TSLP in combination with IL-1β or TNF-α releases several cytokines and chemokines (16, 76, 77). TSLP induces prostaglandin D_2_ (PGD_2_) production by human cord blood-derived cultured mast cells and by human peripheral blood-derived mast cells when combined with IL-33 (78).

*In vivo* administration of TSLP modulates the differentiation of alternatively activated macrophages (79). Interestingly, TSLP synergistically potentiated CCL17 production induced by IL-4 from murine macrophages. The expression of TSLRP and IL-7Rα and the production of TSLP isoforms should be investigated in both human primary macrophages and tumor-associated macrophages.

Human platelets express both TSLPR and IL-7Rα and their activation by TSLP promotes platelet activation (80, 81). Based on these findings, it has been suggested that TSLP could be involved in patients with metabolic syndrome (98) and in atherosclerosis (81, 99).

Wilson and collaborators have demonstrated that activation of protease-activated receptor 2 receptors by serine proteases on keratinocytes can trigger the release of TSLP. This in turn activates TSLP receptor on sensory neurons contributing to itch in patients with atopic dermatitis (AD) (53).

## Short and Long Isoforms of TSLP

Harada and collaborators first identified two variants for TSLP in human bronchial epithelial cells (10). The authors demonstrated that a poly I:C, a toll-like receptor 3 (TLR3) ligand, known to upregulate TSLP (16, 18), induced the upregulation of a long isoform of TSLP. A shorter isoform, composed of 63 amino acids, was constitutively expressed in all normal tissues examined, including human lung fibroblasts, and its expression did not change after challenge with LPS or poly I:C. These two isoforms were initially considered the result of alternative splicing (10). Subsequently, the same group identified two distinct 50-untranslated regions resulting in two different open reading frames for TSLP in the human genome (100). The sequence of the 63 amino acids of short TSLP is homologous to the C-terminus of the long form and specific antibodies anti-sf TSLP are not commercially available. Primer pairs specifically targeting one or the other transcript variant should be used to study the two isoforms of TSLP at the mRNA level. It is important to emphasize that none of all previous studies had used tools to analyze the expression or functions of the two TSLP isoforms. Harada and collaborators also identified two polymorphisms upstream the long isoform untranslated region that increase transcription factor binding and, consequently, long TSLP expression. These two polymorphisms correlate positively with asthma susceptibility, whereas this is not true for the polymorphism found in the second intron of the long form (100). The authors suggested that TSLP could be a therapeutic target in asthma.

Xie et al. studied the differential expression and modulation of the two isoforms in primary human keratinocytes (47). They confirmed that TLR3, TLR2, and TLR6 ligands induced long TSLP in the presence of an atopic cytokine milieu (IL-4, IL-13, and TNF-α). They also reported that the constitutive expression of the short isoform by human keratinocytes is greater than the long form. Cultrone et al. showed that activation of human intestinal epithelial cell lines with cytokines (i.e., IL-1, TNF-α) upregulates long TSLP (14). Another group confirmed that TNF-α activation of primary human lung fibroblasts upregulates the long isoforms (101).

Rescigno and collaborators extensively examined the differential expression and biologic activity of the two isoforms *in vitro* and *in vivo* (15). They confirmed that the two isoforms are not the result of alternative splicing of the same transcript but are rather controlled by two different promoter regions. They also found that in healthy barrier surfaces, such as human intestinal and skin tissue, short TSLP is the main transcript variant. sf TSLP inhibits *in vitro* the production of several cytokines (i.e., TNF-α, IL-1β, IL-6), whereas lf TSLP increases the release of IFN-γ. Importantly, they reported that lf TSLP activates a canonical TSLPR on human immune cells, whereas sf TSLP induces or inhibits signaling through an unknown receptor (15). The authors also found that highly immunogenic strains such as *Salmonella typhimurium* and invasive *Escherichia coli* (*E. coli*) upregulate the long isoform and downregulate the short isoform, whereas the opposite is true after challenge with a commensal *E. coli* strain. The latter observation suggests that the dysbiosis observed in barrier surfaces could impact the expression of TSLP. More recently, Dong et al. reported that inflammatory stimuli upregulate lf TSLP mRNA but not sf TSLP in human bronchial epithelial cells (102). Importantly, administration of sf TSLP decreased airway hyperreactivity and inflammation in a mouse model of asthma. Finally, Kuroda and collaborators found that the constitutive expression of lf TSLP in unstimulated primary human keratinocytes is markeally lower than sf TSLP and that lf TSLP was strongly induced by several allergens (50).

### Short TSLP Functions

Despite increasing evidence of a dichotomy for the two isoforms of TSLP in humans, the physiological role of the short isoform was largely unknown. Bjerkan et al. examined the expression and biologic activity of short TSLP on barrier surfaces such as the oral mucosa and the skin (22). The authors found that TSLP on healthy barriers is limited to the short isoform, whereas the long TSLP is upregulated in mucosal lesions after challenge. Recombinant human long TSLP had previously been found to exert antimicrobial activities (103). Using synthetic overlapping peptides, the authors demonstrated that the antimicrobial effect was primarily mediated by the C-terminal region of TSLP. Bjerkan and collaborators assessed the antimicrobial activity of short TSLP and found that the growth of all bacterial strains tested was markedly inhibited (22). They also addressed the biologic activity of the short isoform *in vitro* by conditioning with short TSLP monocyte-derived DCs, which do not express TSLPR if not activated (104). They found that the anti-inflammatory effect of short TSLP on human monocyte-derived DCs is mediated *via* an as yet unknown mechanism involving p38 phosphorylation (105) rather than STAT5 phosphorylation through which the long isoform signals (15). The latter findings support the hypothesis that sf TSLP activates an unknown receptor different from TSLPR (15).

### TSLP Isoforms in Human Diseases

Rescigno and collaborators have demonstrated the upregulation of long but not short TSLP in patients with AD and ulcerative colitis (15). They also observed a downregulation of the short TSLP transcript in patients with celiac disease (11, 12).

There is compelling evidence that, in human pathologic conditions, TSLP can also be modulated by endogenous proteases. Bianchieri et al. demonstrated that the protease furin, which was upregulated in biopsies from celiac disease patients, can cleave the long isoform producing fragments of 10 and 4 kDa that show different activity on human peripheral blood mononuclear cells compared with the mature TSLP (106). Schleimer and collaborators reported that TSLP is truncated in two fragments (aa 29-124 and aa 131-159) by furin-like and carboxypeptidase N proteases in inflamed tissue. These fragments showed enhanced pro-Th2 activity on mast cells and ILC2 compared with the long TSLP (63, 106).

It would be of great interest to examine the differential expression of the two isoforms in inflammed tissues and peripheral blood from patients with asthma, chronic obstructive pulmonary disease (COPD), EoE, allergic rhinitis, etc. Because of the pathophysiological relevance of long TSLP expression in all these diseases, the cytokine has been repeatedly suggested as a valid target for therapy with antibodies that would target TSLP and/or prevent its binding to TSLPR (42–45). Since the short isoform of TSLP has homeostatic and anti-inflammatory effects, this should be taken into account when designing targeted therapeutic strategies. mAbs used to neutralize TSLP should ideally not interact or hamper the homeostatic functions of short TSLP.

## TSLP and Allergic Inflammation

Genetic analysis has shown an association of polymorphisms in TSLP with several allergic diseases, including asthma and airway hyperresponsiveness, IgE concentrations, and eosinophilia (10, 100, 107, 108). Additional evidence for the relevance of TSLP in airway inflammation has been provided by genetic studies of mice. TSLPR-deficient mice are resistant to the development of ovalbumin-induced inflammation in mice (109, 110). Mice overexpressing TSLP in the airway epithelium develop an inflammatory disease with characteristics of asthma (110). Intranasal delivery of TSLP and antigen leads to the onset of severe disease (111). Asthmatic patients have higher concentrations of TSLP in their lungs (30, 56) and in peripheral blood (112).

### Asthma and Chronic Rhinosinusitis (CRS)

Genetic variants located in or near *TSLP* have been detected in genome wide studies for asthma, rhinitis, and atopy (100, 113–118). Polymorphisms of the *TSLP* gene appear to contribute to Th2-polarized immunity through greater TSLP production by bronchial epithelial cells in response to viral respiratory infections (10, 100, 116). Moreover, TSLP polymorphisms appear to be associated with a higher risk of allergic rhinitis (117).

In a mouse model of asthma microRNA-19b (miR-19b) reduces airway inflammation and remodeling by inhibiting Stat3 signaling through TSLP downregulation (45). Interestingly, anti-TSLP alleviates airway inflammation in a dust mite-induced mouse model of asthma (119). Together, these results suggest that TSLP pathway is a promising target for immunotherapy of bronchial asthma. TSLP mRNA is increased in the airways of severe asthmatic patients (57) and correlates with disease severity (56). TSLP is also increased in bronchoalveolar lavage (BAL) (Liu JACI141:257, 2018), in induced sputum (120), in exhaled breath condensate (121), and in plasma of asthmatic patients (112). Recent evidence suggests that elevated expression of TSLP in the airways is a biomarker of severe refractory asthma (122). Unfortunately, in the above studies, the differential expression of the two isoforms of TSLP was not examined.

**Figure 3:**
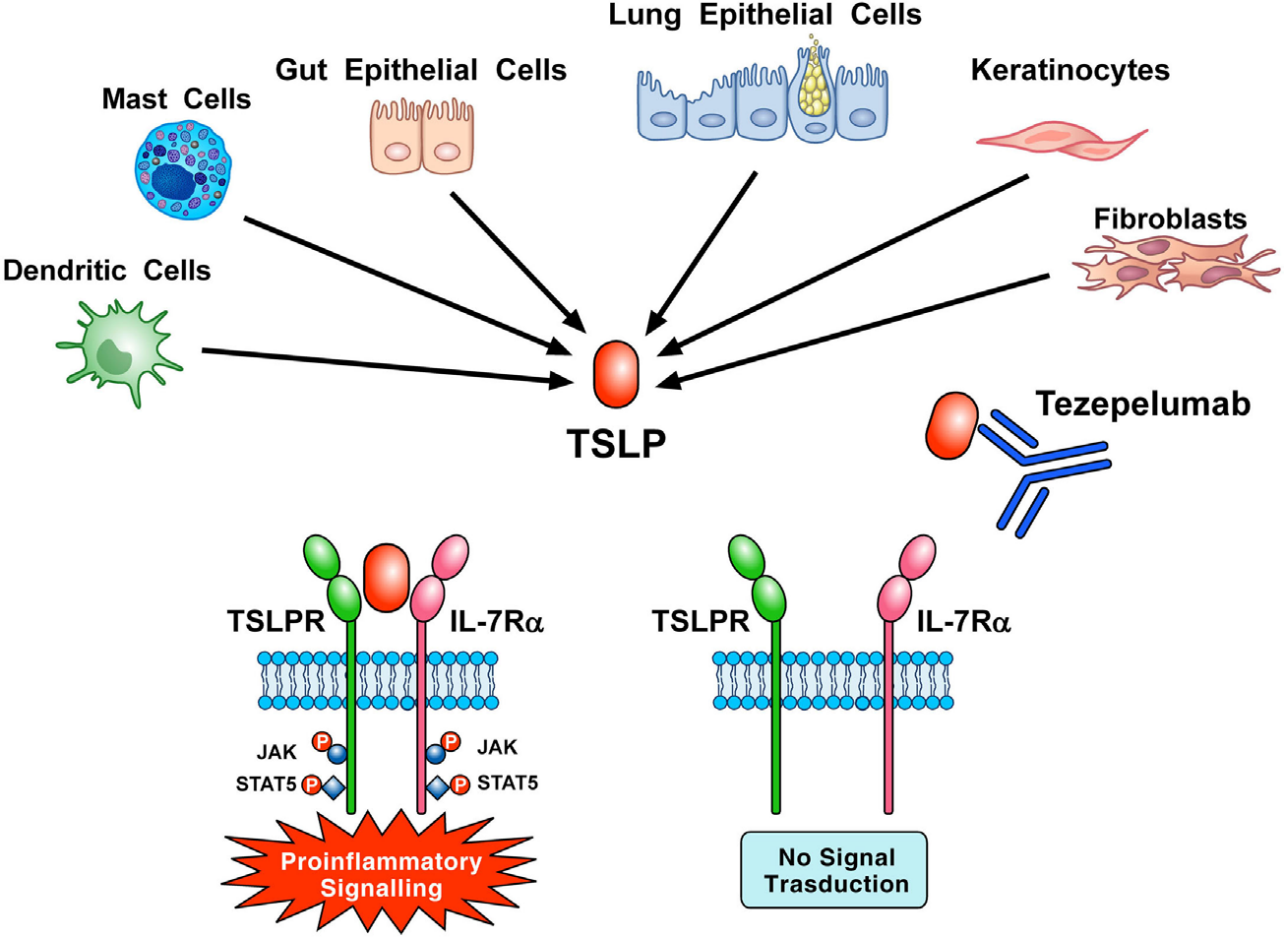
Thymic stromal lymphopoietin (TSLP) produced mainly by gut and epithelial cells and keratinocytes but also by dendritic cells, mast cells, and fibroblasts initiates signaling by establishing a ternary complex with thymic stromal lymphopoietin receptor (TSLPR) and IL-7Rα. Tezepelumab, a human mAb anti-TSLP, binds with high affinity to TSLP and blocks the formation of TSLPR:TSLP: IL-7Rα ternary complex on effector cells. In particular, the variable heavy chain of tezepelumab binds to TSLP, while the variable light chain fragment does not interact with TSLP (44). Tezepelumab inhibits *in vitro* human DC maturation and chemokine production induced by TSLP (44) and reduced exacerbations and improved quality of life in patients with severe uncontrolled asthma (124).

Tezepelumab, a human IgG_2_ mAb (1) anti-TSLP (700 mg i.v. on days 1, 29, and 57) inhibited early and late asthmatic responses, blood and sputum eosinophils, and exhaled nitric oxide in patients with mild, atopic asthma (123). Corren and collaborators reported that tezepelumab (70, 210, or 280 mg s.c. every 4 weeks) reduced asthma exacerbations, blood eosinophils, and Feno and improved FEV_1_ and ACQ-6 score in patients with different asthma phenotypes (124). These two studies indicate that TSLP is an attractive therapeutic target in asthma (Figure 3). Several studies are evaluating the efficacy and safety of tezepelumab alone (NCT 03347279, NCT 03406078, NCT 02698501) or in combination with allergen immunotherapy (NCT 02037196) in asthmatic patients.

Chronic rhinosinusitis is a heterogeneous disease characterized by local inflammation of the upper airways and sinuses (125, 126). Genetic analysis has shown an association of *TSLP* polymorphism with a higher risk for allergic rhinitis (117). TSLP mRNA levels were upregulated in CRS with nasal polyps compared to control subjects and positively correlated with eosinophils and type 2 cytokines (106). The same group of Kato elegantly demonstrated that rh TSLP can be truncated by endogenous serine proteases present in CRS to generate two major peptides which potently activated DCs and ILC2s (63). These results highlight the relevance of posttranslational modifications that control the functional activity of TSLP in human inflammatory disorders. Recent evidence suggests that TSLP controls PGD_2_ production by human mast cells in patients with aspirin-exacerbated respiratory disease (78).

### Eosinophilic Esophagitis

Eosinophilic esophagitis is an emerging disorder distinct from gastroesophageal reflux disease (127, 128). Multiple genome-wide association studies and candidate gene studies have implicated genetic variants of *TSLP* in genetic susceptibility to EoE (129–132). *TSLP* is increased in the esophageal tissue of patients with EoE (129, 130). In a mouse model, EoE-like disease developed independently of IgE, but was dependent on TSLP activation of basophils (92). Interestingly, TSLPR and IL-7Rα are not constitutively expressed by human peripheral blood basophils and TSLP does not activate these cells (9). The latter data apparently contrast with the increased density of basophils in esophageal biopsy of pediatric patients with EoE (92).

### Atopic Dermatitis

Atopic dermatitis is a chronic, inflammatory skin disease affecting up to 15% of children and 2–10% of adults in industrialized countries (133, 134). Genetic variants in *TSLP* are associated with AD (118, 135, 136). TSLP is highly expressed in the lesional skin of patients with AD (25, 40, 95, 137) and TSLP-activated DCs prime naïve T cells to differentiate into Th2 cells (25). Serum levels of TSLP are elevated in AD patients compared to controls (138). However, the relationships between the degree of TSLP expression in the skin and the severity, the phenotypes (i.e., extrinsic *vs* intrinsic), and epidermal barrier function of AD remain to be elucidated (139). Recent evidence indicates that keratinocyte-derived TSLP stimulates pruritus in AD, and perhaps some other dermatologic disorders, by activating TSLP receptor complex on afferent sensory neurons (53). Several clinical trials (NCT02525094, NCT01732510) are evaluating the safety and efficacy of tezepelumab in patients with moderate-to-severe AD.

Basophils are found in skin biopsies of AD patients (134, 140, 141). It has been reported that TSLP elicits basophilia in mice and skin recruitment could be blocked by anti-TSLP antibody suggesting that TSLP promotes skin inflammation through the activation of basophils (74, 141). In a recent study, Voehringer and collaborators have demonstrated in a murine model of AD that skin recruitment of basophils occurred without direct TSLP recognition by basophils (142). Furthermore, basophils did not promote but rather ameliorated skin inflammation, and this effect was dependent on TSLPR expression on murine basophils. Interestingly, human basophils do not express constitutively TSLP and IL-7Rα (9). Together, these results emphasize some of the striking differences between human ad mouse basophils (93, 94).

## TSLP and Chronic Inflammatory Diseases

### COPD and Idiopathic Pulmonary Fibrosis (IPF)

Chronic obstructive pulmonary disease and asthma are conventionally considered T_H_1/macrophage/neutrophils and T_H_/eosinophil/mast cell mediated, respectively (143, 144). TSLP mRNA and protein are overexpressed in the bronchial epithelium of COPD patients compared to controls (30). Several activators of airway epithelial cells such as respiratory viruses (19), double-stranded RNA (17, 18), cigarette smoke extracts (51, 52), and proinflammatory cytokines (16, 20) can stimulate the production of TSLP in COPD patients. TSLP and TSLPR are overexpressed in lung biopsy of patients with IPF (101) and BAL TSLP is increased compared to controls (145).

### Celiac Disease

Celiac disease (CD) is a heterogeneous enteropathy caused in genetically susceptible individuals by the ingestion of gluten (146). TSLPR and IL-7Rα are expressed in CD mucosa and both lf TSLP and sf TSLP mRNAs are reduced in CD compared to control subjects (11). Interestingly, the serin protease furin, which is overexpressed in CD mucosa, degrades lf TSLP. The latter intriguing findings extend the observation that proteases can cleave and modulate the functions of TSLP (63, 106). Collectively, these results indicate that in pathological conditions endogenous proteases can regulate TSLP activities. The role of sf and lf TSLP in experimental inflammatory bowel disease is hampered by the absence of sf TSLP in the mouse (147).

## TSLP and Autoimmune Disorders

The role of TSLP in autoimmune diseases is largerly unknown, and only very few studies have started to explore the role of this pleiotropic cytokine in Th1- or Th17-driven autoimmune disorders. It has been demonstrated that TSLP induced polyclonal B-cell activation *in vitro* and development of autoimmune hemolytic anemia *in vivo* (148). TSLPR-deficient mice showed less severe arthritis in collagen-induced autoimmune arthritis (149). In a model of experimental autoimmune encephalomyelitis (EAE), TSLP-knock-out mice displayed a delayed outset of disease and an attenuated form of EAE (150). These studies suggest that TSLP–TSLPR axis might contribute to the pathogenesis of autoimmune disorders.

### Rheumatoid Arthritis (RA)

Rheumatoid arthritis is a systemic autoimmune disease characterized by chronic synovitis (151). TSLP has been implicated as a possible exacerbating mediator in RA (38). Synovial fluid concentrations of TSLP are increased in RA patients compared to osteoarthritis (39). Fibroblasts from RA patients can produce TSLP when activated by several immunologic stimuli (e.g., IL-1β, TNF-α) (152). In addition, mast cells and macrophages, present in RA synovium (151, 153), may contribute to TSLP levels in the RA joint (28, 56, 154). Supportive role of TSLP in arthritis derives also from several mouse models (38, 149). Blockade of TSLP/TSLPR axis warrants further experimental and clinical studies in RA.

### Psoriasis

Psoriasis is a common, chronic inflammatory disease that manifests predominantly in the skin (155). Although psoriasis is classified as an organ-specific autoimmune disease (156–158), there is increasing understanding of psoriasis as a systemic inflammatory disease that extends beyond the skin (159, 160). The pathophysiology of psoriasis is characterized by skin DC activation and pathogenetic IL-23 production by blood and skin DCs (155). Volpe and collaborators found that TSLP is overexpressed in human psoriatic skin (40). Moreover, they reported that TSLP induces DC maturation and primes for subsequent CD40L-induced IL-23 production by DCs. These original findings extend the role of TSLP from allergic disorders to IL-23-driven autoimmunity, with possible implication in other forms of autoimmune disorders (e.g., RA).

## TSLP and Cancer

Initially shown to promote the growth and activation of B cells and DCs (3, 25), TSLP is now known to have wide-ranging effects on cells of innate and adaptive immune system (Figure 2). These include DCs, ILC2, T and B cells, NKT and Treg cells, eosinophils, neutrophils, basophils, mast cells, and macrophages. All these cells are implicated in tumor initiation and growth, angiogenesis, and lymphangiogenesis (161–163). Therefore, it is not surprising that TSLP plays a direct and/or indirect role in the control of a variety of experimental and human cancers (34).

A pro-tumorigenic role of TSLP is supported by a study using an orthotopic model of breast cancer in the mouse (164). Using a variety of methods, several groups found that genetic rearrangements and mutations in the *TSLP* gene are present in pediatric acute lymphoblastic leukemia (165–167). TSLP is overexpressed in plasma and lymph nodes from Hodgkin patients (41). Moreover, TSLP acts through the production of Th2 cytokines (e.g., IL-4 and IL-13) to induce cutaneous T-cell lymphoma (168). De Monte and collaborators elegantly demonstrated that human pancreatic CAF release TSLP which activates TSLPR^+^ DCs to drive Th2 differentiation mediated by the release of IL-4 from basophils (32). It has been reported that human cervical carcinoma cells release TSLP acting on TSLPR^+^ endothelial cells to promote angiogenesis and cancer growth (169). In another study, it was reported that breast cancer cells can produce TSLP (170). Kuan and Ziegler extended the previous observation demonstrating that TSLP promotes the survival of breast cancer cells through the expression of the antiapoptotic molecule Bcl-2 (171).

In contrast to these studies, two groups have demonstrated a tumor-suppressing role for TSLP in murine models of skin carcinoma (172, 173). Yue and collaborators found decreased TSLP expression in human colon cancer and TSLP levels negatively correlated with the clinical staging score of cancer (174). Moreover, TSLP enhanced apoptosis of colon cancer cells through the engagement of TSLPR. Finally, using a xenograft mouse model, the authors found that peritumoral administration of TSLP reduced tumor growth. In mouse models of breast and pancreatic carcinogenesis, it was found that early administration of TSLP blocked cancer development. The antitumor effect of TSLP was mediated by activation of CD4^+^ Th2 cells around tumors and in draining LNs (175). Finally, Soumelis and collaborators reported that TSLP was undetectable or expressed at low levels in breast cancer and in several human breast cancer cell lines (176).

In conclusion, the role of TSLP–TSLPR axis in experimental and human cancer is still controversial (34). In certain neoplasias, TSLP plays a pro-tumorigenic role, whereas in others, a protective role. It is obvious that there are many important questions that should be urgently addressed before we understand whether TSLP isoforms and TSLPR^+^ immune cells are an ally or an adversary in different types of human cancer. This is another fundamental question that should be seriously considered when designing TSLP-targeted therapeutic strategies. Finally, studies are urgently needed to examine the differential expression and functions of sf TSLP and lf TSLP on patients with cancer.

## Conclusion and Perspectives

Thymic stromal lymphopoietin is a cytokine originally characterized by its ability to promote DC and B cell activation (177). There is increasing evidence that TSLP can directly and/or indirectly activate a plethora of immune and non-immune cells involved in a wide spectrum of inflammatory disorders and cancer (161–163). These emerging observations greatly widen the role of TSLP in different human diseases.

It is important to emphasize that several groups have demonstrated the existence of two variants (sf and lf TSLP) of TSLP in humans (10, 14, 15, 22, 47, 100, 101). sf TSLP is constitutively expressed in steady state in different human tissues and plays a homeostatic role (15). By contrast, lf TSLP is induced at sites of inflammation and plays a proinflammatory role (14, 15, 47, 50, 100, 102). lf TSLP has a sequence of 159 amino acids, which corresponds to the commercially available recombinant TSLP produced by prokaryotic cells. Moreover, sf TSLP overlaps the lf TSLP in the terminal region and available anti-TSLP antibodies do not distinguish between the two isoforms. Therefore, at present, the use of specific primers is the only way to distinguish the two TSLP variants at the molecular level. Unfortunately, only few studies have examined the expression and functions of the two TSLP variants in different human disorders. The studies examining the two isoforms have already revealed interesting dichotomies in the expression and function of two TSLP variants in different inflammatory diseases (11, 14, 15, 22, 100). Additional studies are urgently needed to evaluate the presence and function of the two isoforms of TSLP in different pathological conditions.

An additional level of complexity derives from the observation that, in pathological conditions, TSLP can be cleaved by several endogenous proteases (11, 63, 106). The latter observation is not unexpected because another epithelial-derived cytokine such as IL-33 can be cleaved by several endogenous (i.e., tryptase) (178) and exogenous proteases (179).

The role of TSLP in different types of cancer is controversial (34, 171, 174). The role of the two TSLP isoforms has not been investigated in most of these studies. In addition, the commercial TSLP assay used in some studies (41) does not distinguish the two TSLP isoforms. The role of the two isoforms of TSLP in human cancers should be taken into account in future studies.

Given the role of TSLP in experimental and clinical asthma (45, 56, 57, 119, 180) and in AD (25, 40, 95, 137), this cytokine soon appeared an attractive therapeutic target (100). Tezepelumab is a first-in-class human mAb that binds to TSLP inhibiting its interaction with TSLP receptor complex (44). Tezepelumab given as an add-on therapy to patients with severe uncontrolled reduced asthma exacerbations and improved quality of life (124). It is unknown whether tezepelumab administration in humans affects plasma levels or the bronchial expression of the two TSLP variants. Several undergoing clinical trials are investigating the efficacy of tezepelumab in AD. Because the short form of TSLP has important homeostatic (15) and antibacterial activities (22), the long-term safety should be taken into account when designing anti-TSLP targeted therapeutic strategies.

Thymic stromal lymphopoietin was initially considered a key cytokine that initiates and promotes type 2 immunity (177). Increasing evidence suggests that TSLP could mediate immune inflammation also in patients with autoimmune disorders such as RA (38, 39) and psoriasis (40). The latter findings add to the complexity of TSLP interactions with several immune cells. We would like to suggest that TSLP and/or perhaps TSLP isoforms might have different immunomodulatory roles, depending on the type of immune environment.

Given the complexity of the interactions of different isoforms and cleavage products of TSLP with a plethora of immune cells in various organs and in different diseases, several fundamental questions remain to be elucidated. A better understanding of the impact of TSLP isoforms and cleavage products on immune cells will be critical for the development of safe and effective TSLP targeted therapies in inflammatory disorders and cancer.

## Author Contributions

All authors contributed equally to reviewing the current literature and writing of the manuscript.

## Conflict of Interest Statement

The authors declare that the research was conducted in the absence of any commercial or financial relationships that could be construed as a potential conflict of interest.

## Abbreviations

AD, atopic dermatitis; ALL, acute lymphoblastic leukemia; ASM, airway smooth muscle; BAL, bronchoalveolar lavage; CD, celiac disease; COPD, chronic obstructive pulmonary disease; CRS, chronic rhinosinusitis; DC, dendritic cell; dsRNA, double-stranded RNA; *E. coli, Escherichia coli*; EoE, eosinophilic esophagitis; γc, γ chain; GERD, gastroesophageal reflux; GWAS, genome-wide association studies; ILC, innate lymphoid cell; lfTSLP, long form of TSLP; LN, lymph node; LPS, lipopolysaccharide; mAb, monoclonal antibody; PAR2, protease- activated receptor 2; Poly I: C, polyinosinic-polycytidylic acid; PGD_2_, prostaglandin D_2_; RA, rheumatoid arthritis; sfTSLP, short form of TSLP; Tfh, T follicular helper; Th2, T helper type 2; TLR, toll like receptor; Treg, T regulatory; TSLP, thymic stromal lymphopoietin; TSLPR, thymic stromal lymphopoietin receptor.
